# Every non–obstructive hydrocephalus is not due to tuberculous basal meningitis

**DOI:** 10.4103/0972-2327.74191

**Published:** 2010

**Authors:** Deepak Goel, K. K. Bansal, Manish Mittal, Ranjeet

**Affiliations:** Himalayan Institute, Swami Ram Nagar, Doiwala, Dehradun, India

## Introduction

**Figure 1:**
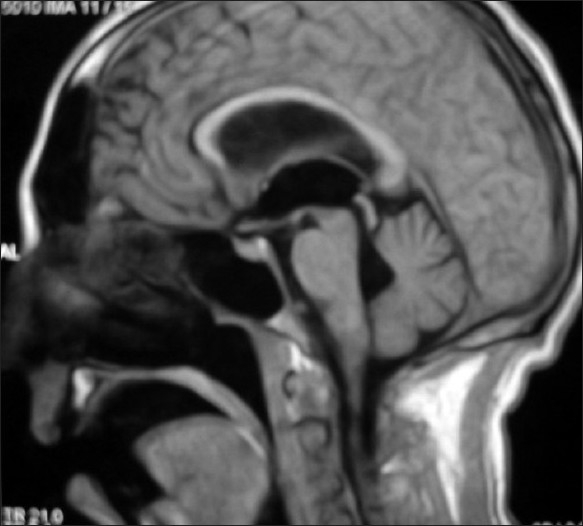
T1W sagittal MRI showing no obvious obstruction

**Figure 2:**
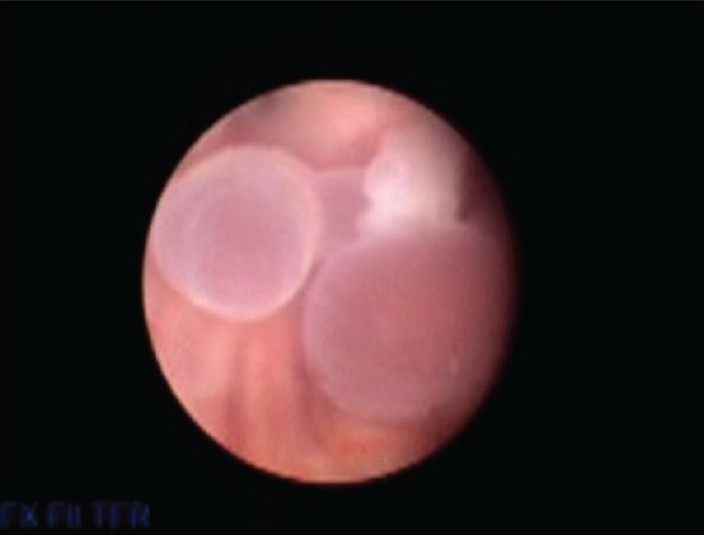
Endoscopic view showing two cysticerci in 4th ventricle

A 42 year male presented with episodic headache and vomiting. Two years earlier he had similar symptoms when a diagnosis of tuberculous meningitis was made after CSF examination. He was treated with antitubercular drugs and a ventriculoperitoneal shunt was placed with good relief. One year later he had recurrence of headache when second scan showed persistent of hydrocephalus [Figure 1]. Considering a shunt failure, a shunt revision surgery was done without much relief. We proceeded to carry out an endoscopic third ventriculostomy (ETV) To our surprise, after third ventriculostomy as the endoscope was negotiated into aqueduct, two grapes like structures were visualized that were attached to the floor of 4th ventricle [Figure 2]. En-block removal of the cysts could be done and diagnosis of neurocysticercosis (NCC) was confirmed.

In India, both NCC and tuberculosis of central nervous system (CNS) are common endemic problems. Obstructive hydrocephalus by CNS tuberculosis is common,[1] but hydrocephalus due to intraventricular NCC is reported in case series or case reports only.[2] One author reported that intraventricular NCC constitutes only 7-20% of all cases affected by this infestation.[3]

It is important to consider that hydrocephalus in the absence of obvious obstruction might not be due to CNS tuberculosis. New imaging such as three-dimensional constructive interference in steady state (3D-CISS) or heavily T2W sequences could improve the diagnosis.[4] In cases where hydrocephalus is acute in the onset and tubercular toxemia is absent, ETV is better choice. Moreover, in cases of shunt failure, ETV can be a better choice than revision of shunt.[3]
